# Ve-ptp Modulates Vascular Integrity by Promoting Adherens Junction Maturation

**DOI:** 10.1371/journal.pone.0051245

**Published:** 2012-12-14

**Authors:** Silvia Carra, Efrem Foglia, Solei Cermenati, Erica Bresciani, Costanza Giampietro, Carla Lora Lamia, Elisabetta Dejana, Monica Beltrame, Franco Cotelli

**Affiliations:** 1 Dipartimento di Biologia, Universitàdegli Studi di Milano, Milan, Italy; 2 Dipartimento di Bioscienze, Universitàdegli Studi di Milano, Milan, Italy; 3 Dipartimento di Scienze Biomolecolari e Biotecnologie, Universitàdegli Studi di Milano, Milan, Italy; 4 FIRC Institute of Molecular Oncology, Milan, Italy; Katholieke Universiteit Leuven, Belgium

## Abstract

**Background:**

Endothelial cell junctions control blood vessel permeability. Altered permeability can be associated with vascular fragility that leads to vessel weakness and haemorrhage formation. *In vivo* studies on the function of genes involved in the maintenance of vascular integrity are essential to better understand the molecular basis of diseases linked to permeability defects. Ve-ptp (Vascular Endothelial-Protein Tyrosine Phosphatase) is a transmembrane protein present at endothelial adherens junctions (AJs).

**Methodology/Principal Findings:**

We investigated the role of Ve-ptp in AJ maturation/stability and in the modulation of endothelial permeability using zebrafish (*Danio rerio*). Whole-mount *in situ* hybridizations revealed z*ve-ptp* expression exclusively in the developing vascular system. Generation of altered z*ve-ptp* transcripts, induced separately by two different splicing morpholinos, resulted in permeability defects closely linked to vascular wall fragility. The ultrastructural analysis revealed a statistically significant reduction of junction complexes and the presence of immature AJs in z*ve-ptp* morphants but not in control embryos.

**Conclusions/Significance:**

Here we show the first *in vivo* evidence of a potentially critical role played by Ve-ptp in AJ maturation, an important event for permeability modulation and for the development of a functional vascular system.

## Introduction

The vascular endothelium plays a physiological role as a selective barrier between blood and extravascular tissues and it is involved in the formation and in the maintenance of vascular structures. The performing of these important functions is closely related to the regulation of endothelial cell-cell adhesions [1], [2].

Endothelial cells (ECs) contact each other by specialized junctional regions which are comparable to Adherens Junctions (AJs) and Tight Junctions (TJs) that are present in the epithelial tissues. These junctions are formed by different transmembrane adhesive proteins that bind, with their cytoplasmic domain, to intracellular partners anchoring them to cytoskeletal filaments [3]. In AJs the adhesion is mediated, in part, by the transmembrane protein VE-cadherin, which forms pericellular zipper-like structures along cell-cell contacts [3]–[6].

The failure of correct intercellular EC adhesion leads to a reduced control of permeability and altered vascular morphogenesis. This may be the cause of important human pathologies which include vascular malformations [7], [8], stroke, edema or metastatic spread of tumors [9].

The functionality and the integrity of cell-cell junctions are affected by the phosphorylation level of adhesion proteins or of their associated components. Many phosphatases and kinases are directly or indirectly associated with AJs components and the balance between the Protein Tyrosine Phosphatases (PTPs) and kinases activity is critical for the regulation of junctional stability [4], [10], [11]. A number of *in vitro* studies support a tight relationship between the role of PTPs and the maintenance of EC-EC junctional integrity and endothelial barrier function, showing that high phosphorylation levels promote junctional disassembly and the opening of a paracellular pathway [12]–[17].

Ve-ptp is a specific Vascular Endothelial-Protein Tyrosine Phosphatase, which is exclusively expressed in endothelial cells [18]–[20]. Mouse *Ve-ptp* encodes a 200 kDa polypeptide [21], composed of 17 extracellular fibronectin type III-like repeats (FN3), one transmembrane domain and one intracellular phosphatase domain (PTP domain) [21]. *Ve-ptp* mutants and null-mice die by E9.5 for severe angiogenic and vascular remodeling defects [18], [19].

The structure of the Ve-ptp extracellular domain suggests a role of this protein as an adhesion receptor. *In vitro* studies have shown that Ve-ptp and VE-cadherin interact through their extracellular domains and this interaction modulates the VE-cadherin phosphorylation level affecting vascular permeability [21]–[25]. A recent study has demonstrated in mouse that the dissociation of Ve-ptp from VE-cadherin is a prerequisite for the destabilization of EC contacts and for the opening of endothelial junctions [26]. Furthermore *in vitro* data have demonstrated a role of Ve-ptp in fine-tuning the activity of two tyrosine kinases which play an important role in vascular morphogenesis and in angiogenic/remodeling processes such as Tie-2 and Vegfr2 [27], [28].

In our work we could demonstrate the involvement of Ve-ptp in the control of endothelium integrity and consequently its role in the modulation of vascular permeability *in vivo*, using zebrafish as a model system. In order to shed light on the structural role of Ve-ptp in the EC junctions, we performed loss-of-function experiments by injecting separately two independent splice-blocking morpholinos that produced two altered forms of z*ve-ptp* transcript. This approach allowed us to assess the effects on vascular stability of a zVe-ptp predicted altered protein lacking part of the extracellular domain. Our data point to an involvement of Ve-ptp in the maturation of AJs and to the important role played by this protein in the vascular stability.

## Results

### Identification and sequence analysis of z*ve-ptp* gene

**Figure 1:**
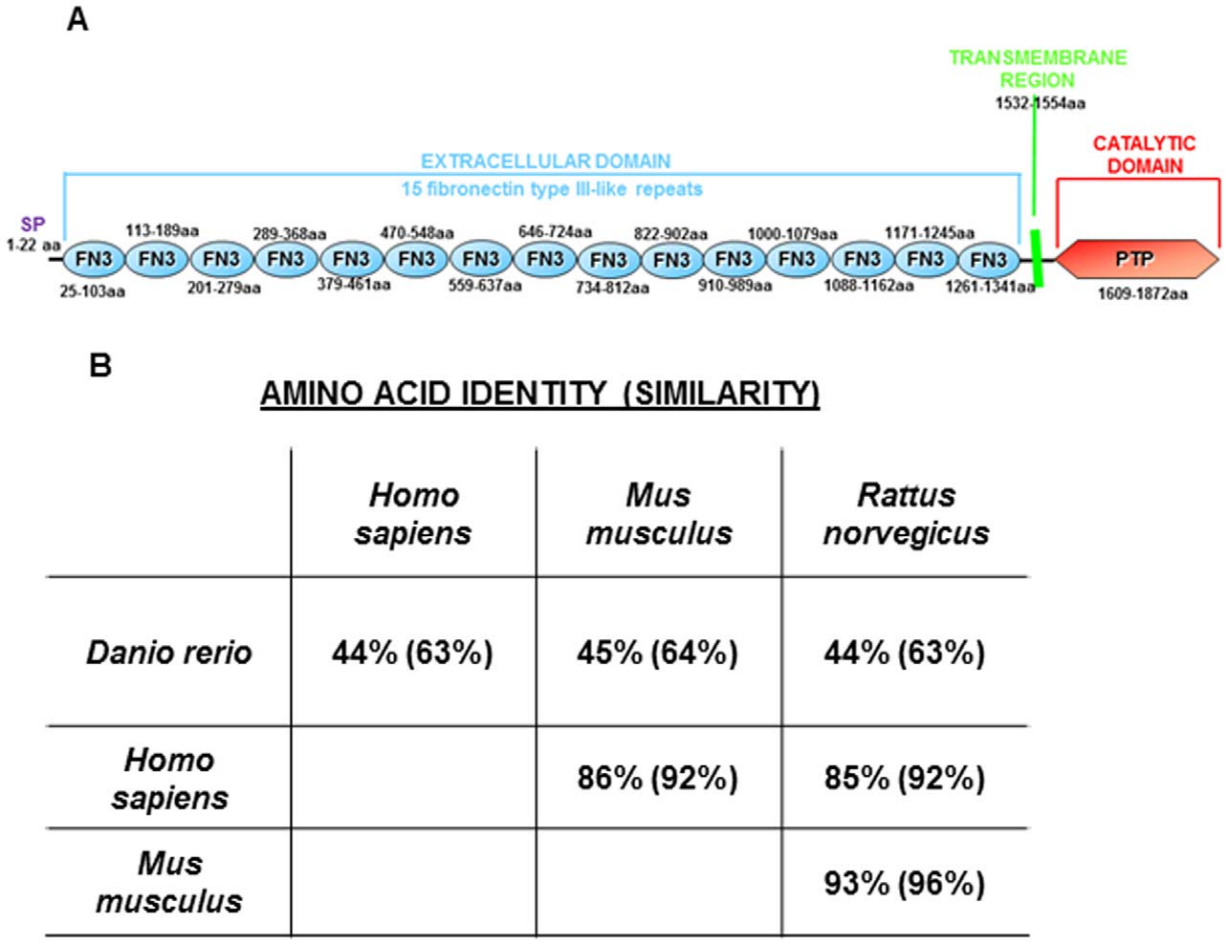
The zVe-ptp protein. (A) Schematic representation of the zVe-ptp domain structure. The protein is composed of an intracellular domain (red), a transmembrane region (green) and 15 fibronectin type III-like repeats (light blue). The amino acid residue (aa) numbers of each domain are indicated. SP: signal peptide; FN3: fibronectin type III-like repeat; PTP: tyrosine phosphatase domain. (B) Table of amino acid identity (and similarity) between zVe-ptp protein and its human, rat and mouse orthologs. The table is the result of multiple alignment of proteins using the VECTOR NTI version 10.0 (Invitrogen) program and the following sequences: *Homo sapiens* (gi: 75517206); *Mus musculus* (gi: 23618914); *Rattus norvegicus* (gi: 109480532).

We identified the zebrafish *ve-ptp* sequence (GenBank FJ435363) using the combination of EST and genomic databases screening and several 5′ RACE steps. The putative z*ve-ptp* cDNA sequence is 6.4 kb long. The 5.7 kb open reading frame encodes a protein of 1892 amino acids. The zVe-ptp protein sequence was scanned for conserved protein domains. This analysis revealed that the deduced zebrafish Ve-ptp protein sequence is composed of a signal peptide, 15 fibronectin type III-like (FN3) repeats, a transmembrane region and a unique PTP domain in the intracellular region (Figure 1 A). Therefore, the structure of the *zve-ptp* gene product is very similar to that of mouse Ve-ptp, suggesting that Ve-ptp is a receptor-type protein with a tyrosine phosphatase activity also in zebrafish. The comparison between zVe-ptp protein and its human/rat and mouse orthologs revealed, respectively, 44% and 45% amino acid identity, and 63% and 64% amino acid similarity (Figure 1 B).

### z*ve-ptp* is expressed in the developing vascular system

**Figure 2:**
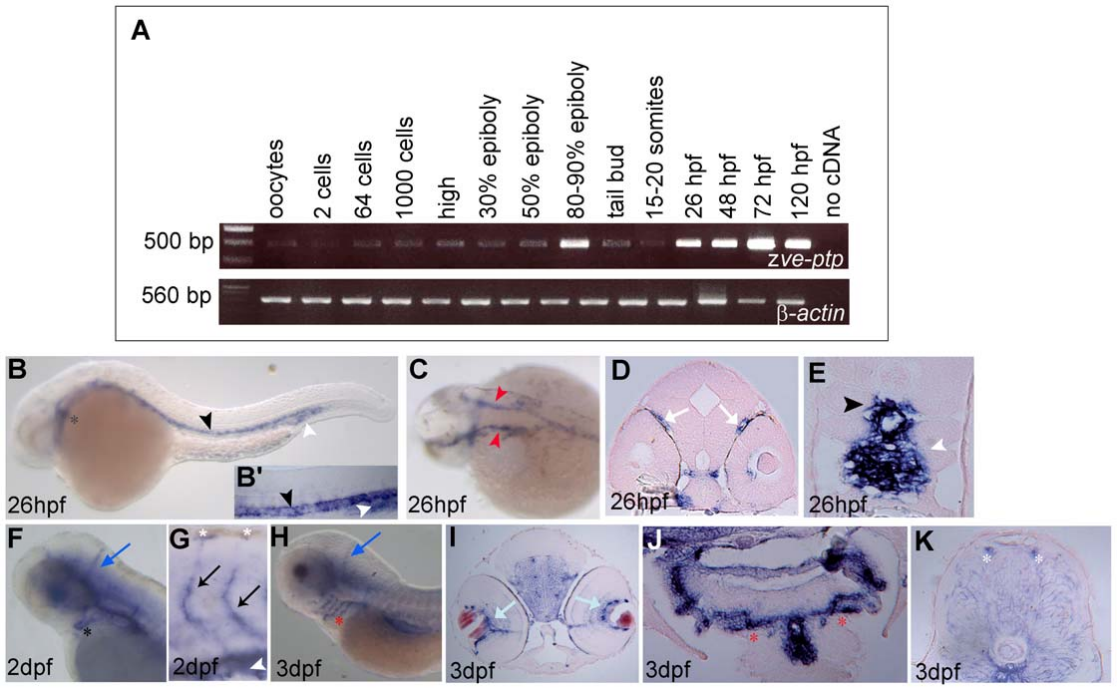
Spatio-temporal expression pattern of z*ve-ptp*. (A) z*ve-ptp* and *β-actin* temporal expression analysis is performed by qualitative RT-PCR analysis on total RNAs extracted from oocytes and different embryonic and larval stages (from 2 cells to 120 hpf). Negative control is showed in the right-most line. The sizes of the obtained PCR fragments are indicated. z*ve-ptp* is expressed both maternally and zygotically during zebrafish development. (B–K) The z*ve-ptp* spatial expression was analyzed by WISH at various developmental stages. Lateral (B) and dorsal (C) view of a 26 hpf embryo with anterior to the left; (B′) higher magnification of the tail shows the expression in the DA and CV. The transverse sections at the level of the head (D) and the tail (E) of a 26 hpf embryo labelled with z*ve-ptp* probe show in detail the signal in head vessels and in the DA and CV. Lateral views of head (F) and magnification of the tail (G) of a 2 dpf embryo and lateral view of the head of a 3 dpf embryo (H). Cross sections at the level of head (I), aortic arches (J) and trunk (K) of a 3 dpf embryo. Black asterisk: AA1; black arrowhead: DA; white arrowhead: CV; red arrowhead: LDA; white arrows: vessels around the eyes; blue arrows: cranial vessels; black arrow: Se; red asterisks: aortic arches; light blue arrows: vessels around the lens; white asterisks: DLAVs.

z*ve-ptp* transcripts are both maternally and zygotically expressed as deduced from our qualitative RT-PCR analysis (Figure 2 A). Whole-mount *in situ* hybridization analysis revealed that z*ve-ptp* is specifically expressed in the developing vascular system of zebrafish embryos and early larvae (Figure 2 B–K). At 26 hpf (hours post fertilization), z*ve-ptp* expression is predominantly visible in the aortic vessels (Figure 2 B, C), as described at the earliest stage analyzed in mouse (E9.5) [20]. In the head, z*ve-ptp* is expressed in the right and left lateral dorsal aortae (LDA, Figure 2 C) and the first mandibular arch (AA1; Figure 2 B). Histological sections at the head level revealed also a periocular signal in the cranial vasculature (Figure 2 D). The z*ve-ptp* gene is expressed in the whole dorsal aorta (DA) along trunk and tail region (Figure 2 B, B′). At this stage and at 2 dpf (days post fertilization), a strong z*ve-ptp* hybridization signal was detectable in the caudal region of the axial vein (CV; Figure 2 B, B′, E). Starting from 2 dpf, z*ve-ptp* expression is observed mainly in vessels of small size such as in the developing cranial vasculature, intersegmental vessels (Se) and at 3 dpf in the complete set of the aortic arches (Figure 2 F–J) and in the dorsal longitudinal anastomotic vessels (DLAVs, Figure 2 K). In conclusion, the expression pattern of zebrafish *ve-ptp* is closely similar to that of mouse *Ve-ptp* [18]–[20].

### The injection of z*ve-ptp* morpholinos causes defects in angiogenesis and in vascular integrity

To analyze the role of Ve-ptp during zebrafish early development, we performed loss-of-function experiments injecting three different morpholinos. The injection of different amounts of each MO led to morphological defects and increased mortality in a dose dependent manner (Figure S1). The first z*ve-ptp* MO (MOa) is designed to block mRNA translation. The injection of the selected dose of 0.5 pmol/embryo of MOa in the tg(*fli1*:EGFP)^y1^ line [29] led mainly to circulatory and angiogenic defects, without substantially affecting embryo morphology at 2 dpf. The main axial vessels appeared grossly normal in all injected embryos, even if z*ve-ptp* MOa morphants presented the reduction or the complete absence of circulation (68%; n = 56). The main vascular defects observed were in the intersomitic vessels of the tail (71%; n = 56), which appeared truncated or with anomalous branching (Figure S2 B). These data are in agreement with the *Ve-ptp* mutant and null mice phenotypes, confirming in zebrafish the conservation of Ve-ptp function in angiogenic and remodeling processes [18], [19].

**Figure 3:**
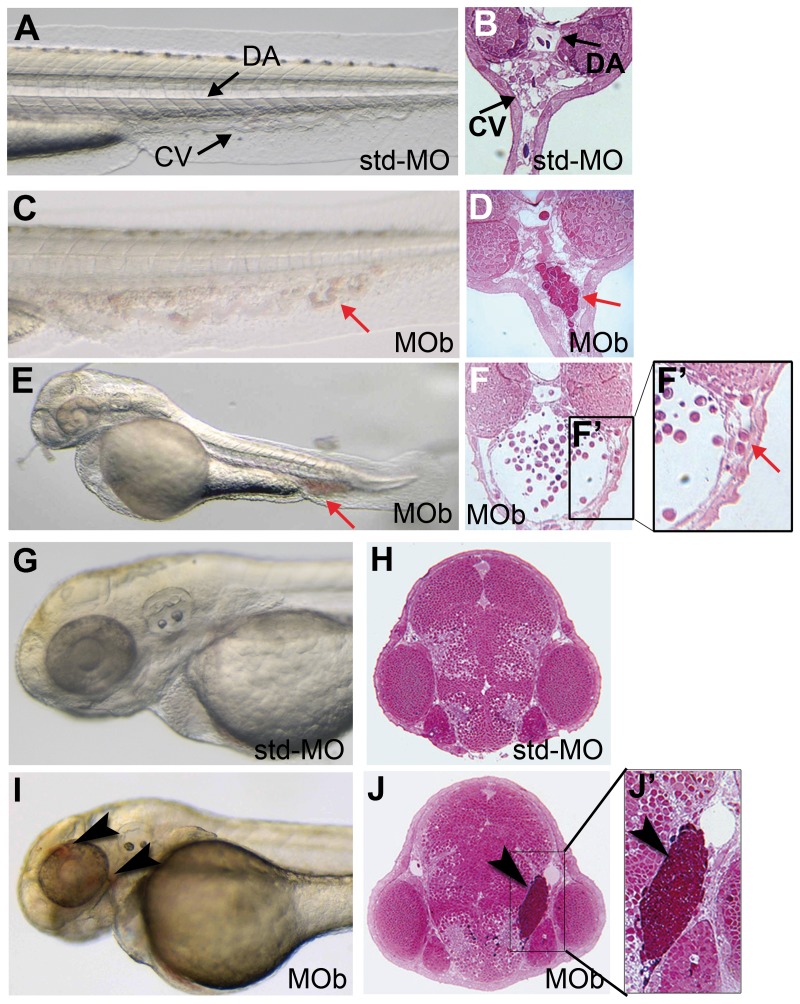
The z*ve-ptp* MOb injection caused blood cell aggregates in the CV and head haemorrhages at 2 dpf. (A) Bright-field image and (B) cross section of the tail of a control embryo injected with std-MO. (C) Bright-field image and (D) cross section of the tail of an embryo injected with MOb showing blood aggregates in CV (red arrow). (E) Lateral view and (F) cross section at the level of the CV of a MOb injected embryo with a severe phenotype; (F′) higher magnification of the boxed area in F showing some blood elements out of the CV. (G) Bright-field image and (H) cross section of the head of a std-MO injected embryo. (I) Bright-field image and (J) cross section of the head of an embryo injected with MOb showing small haemorrhages (black arrowhead). (J′) Higher magnification of the boxed area in J showing blood accumulation in the tissues around the eye of z*ve-ptp* morphant. Anterior to the left. DA: dorsal aorta; CV: caudal vein; red arrow: blood aggregates; black arrowhead: haemorrhage.

To shed light on the role played by Ve-ptp in ECs adhesion *in vivo*, we decided to inject two different splice-blocking morpholinos, z*ve-ptp* MOb (MOb) and z*ve-ptp* MOc (MOc), which putatively target only the zVe-ptp extracellular domain without directly impairing the intracellular catalytic domain. In fact, the coding sequence of z*ve-ptp* remains in frame downstream of the MOs-induced RNA processing alterations. MOb injection resulted in the production of an additional aberrant transcript encoding a protein lacking the 11th FN3 repeat encoded by exon 12 (Figure S2 E). MOb injection in the tg(*fli1*:EGFP)^y1^ line caused no evident angiogenic/remodeling abnormalities at 2 dpf (Figure S2 C). At this stage, embryos injected with the selected dose of 0.5 pmol showed mainly circulatory and vascular defects (Figure S1 B). The most evident defects in the axial vessels were detected at the level of the CV plexus. About 50% (n = 113) of z*ve-ptp* MOb morphants presented blood cell accumulations in the CV region (Figure 3 C, D). Histological sections revealed that these blood aggregates were present within the blood vessels (Figure 3 D). In a large number of z*ve-ptp* MOb morphants, these cells exhibited only a slight pulsatile motion *in vivo* and the circulation was not completely blocked (movie S2), suggesting that there could be a loss of blood fluidity due to fluid extravasation. In fact, embryos with the most severe phenotype were characterized by the presence of a huge blood stasis in the CV associated with the total loss of circulation (Figure 3 E, F). A limited percentage of these morphants showed a short tail and defects in the central nervous system (9%; Figure 3 E). In the vast majority of these z*ve-ptp* morphants, the CV plexus was severely disorganized and in some cases morphants showed some blood elements out of the vein suggesting a leakage in the vessel wall (Figure 3 F, F′). These defects, closely linked to the loss of endothelial integrity, are supported by the presence of small haemorrhages in the head region in 10% of z*ve-ptp* MOb morphants (Figure 3 I, J; Figure S1). The injection of the second splice-blocking MO, z*ve-ptp* MOc, gave qualitatively similar results to MOb injection, though with a different penetrance (Figure S1 C). z*ve-ptp* MOc targeted the intron 12/exon 13 boundary and its injection resulted in the production of an additional aberrant transcript encoding a protein lacking the 12th FN3 repeat encoded by exon 13 (Figure S2 F). At 2 dpf embryos injected with the selected dose of 0.2 pmol of MOc in combination with 0.3 pmol of p53 MO, to reduce non-specific defects in the central nervous system, showed haemorrhagic phenotypes (21%; n = 53; Figure S1 C; Figure S3 C) and the presence of microstases in the CV plexus (18%; n = 53; Figure S1 C; Figure S3 D). These results indicate that permeability defects and vascular fragility are specifically linked to the predicted impairment of the zVe-ptp extracellular domain.

### The impairment of zVe-ptp extracellular domain results in increased vascular permeability associated with endothelial AJ disorganization

**Figure 4:**
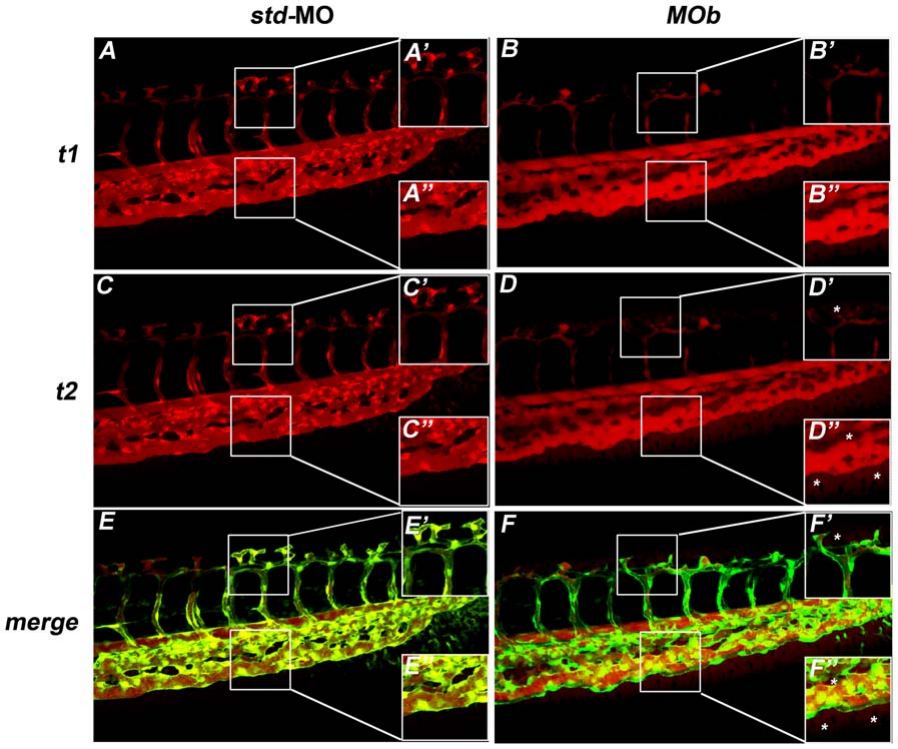
The z*ve-ptp* MOb injection caused an increase in vascular permeability. (A–D) Microangiographies were performed on tg(*fli1*:EGFP)^y1^ embryos at 2 dpf by the injection of dextran-TMR (tetramethylrhodamine; molecular weight 70 kDa). All microinjected embryos presented blood circulation. Confocal images of tail vessels of std-MO (A, C) and MOb injected embryos (B, D) at t1 = 10 minutes (A, B) and t2 = 15 minutes (C, D). (E, F) Merge of the images at t2 of embryos injected with std-MO and MOb with the respective images of the tail vessels obtained using tg(*fli1*:EGFP)^y1^ line. (A′–F′; A″–F″) Higher magnifications of the respective boxed areas. Asterisks: dye extravasation.

The *in vivo* analysis of MOb and MOc morphant phenotypes prompted us to directly investigate the effect of z*ve-ptp* MOs injection on endothelial permeability. We focused our attention on the CV plexus, the region that showed the vast majority of the vascular defects observed in z*ve-ptp* morphants. Microangiography experiments were performed injecting dextran-TMR (tetramethylrhodamine; molecular weight 70 kDa) at 2 dpf only on morphants morphologically unaffected and with intact circulation. These experiments showed that the main axial vessels were lumenized and the circulation was substantially unaffected in all injected embryos. The analysis was carried out at two different time points after injection (t1 = 10 min; t2 = 15 min) when no substantial dye extravasation was detectable in controls. Only 38% of control embryos (n = 29) displayed dye extravasation in the tail region indicative of the presumably physiological outflow. On the contrary, about 80% of the analyzed z*ve-ptp* MOb morphants (n = 32) showed a leakage of the dye out of the CV and the DLAVs (Figure 4 D, D′, D″, F, F′, F″), pointing out the crucial role played by zVe-ptp in the modulation of vascular permeability. We performed the same analysis on z*ve-ptp* MOc and MOa injected embryos. We obtained qualitatively similar results in embryos injected with MOc (61% of dye extravasation; n = 20; Figure S4). Unfortunately, a relevant number of MOa morphants displayed severe circulatory defects preventing us from performing microangiography on these embryos. MOa morphants with intact circulation did not display a significant increase in the percentage of dye leakage (17%; n = 18; Figure S4 G) with respect to control embryos (11%; n = 9; Figure S4 G).

**Figure 5:**
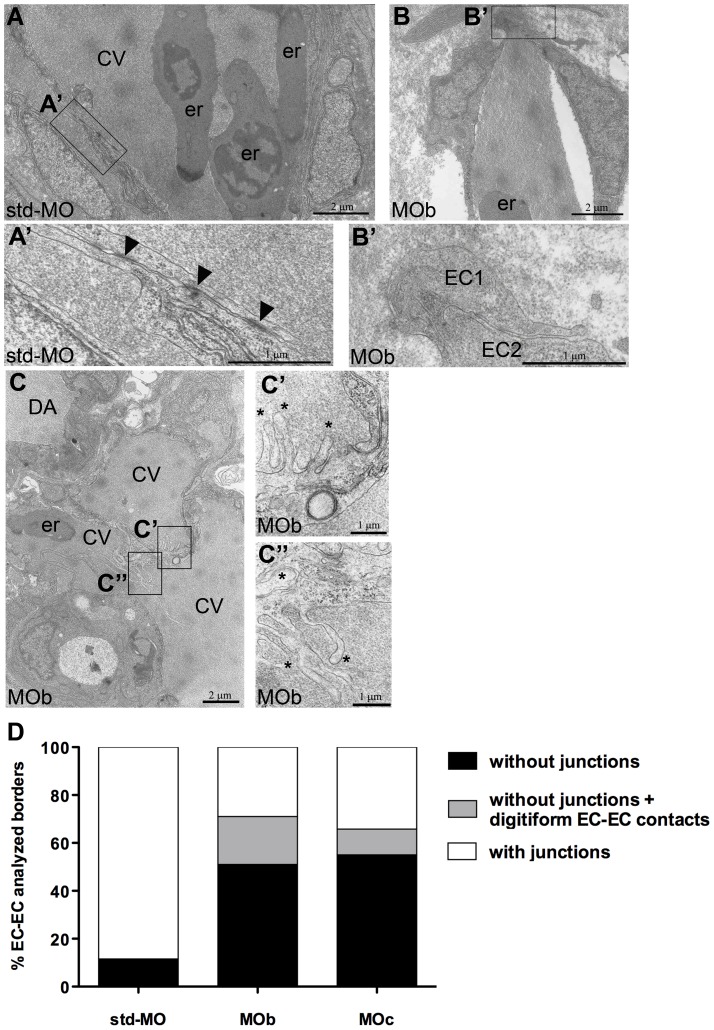
Ultrastructural defects in endothelial cells of z*ve-ptp* morphants at 2 dpf. (A–C) Electron microscopy at the level of the CV plexus of a std-MO (A) and a MOb injected embryo (B, C). (A′–C′, C″) Higher magnifications of the respective boxed areas. (A′) Numerous and extensive junctional complexes are visible in a control embryo. (B′) No junctional complexes are detectable between two ECs in a morphant. All analyzed embryos presented blood circulation. Arrowheads: junctional complexes; asterisks: digitiform EC-EC contact; CV: caudal vein; er: erythrocytes; DA: dorsal aorta; EC: endothelial cell. (D) Quantitative analysis of % EC-EC borders with any type of junctions (adherens and/or tight junctions), without junctions and without junctions but with digitiform EC-EC contacts in controls and in z*ve-ptp* morphants. The graph shows the analysis performed on the acquired images of trunk and tail regions of MOb and MOc injected embryos. We analyzed 112, 150 and 280 EC-EC borders out of three std-MO, seven z*ve-ptp* MOb and five z*ve-ptp* MOc independent injected embryos respectively.

To gain insight into the vascular ultrastructure of z*ve-ptp* morphants, we decided to use a transmission electron microscopy (TEM) approach. We focused our attention on tail vessel ECs of z*ve-ptp* morphants and std-MO injected embryos at 2 dpf (Figure 5). Normal blood vessels are formed by flat endothelial cells that contact each other by numerous and extensive junctional complexes, which appear as well-organized structures (Figure 5 A′). In all z*ve-ptp* morphants, we primarily detected a statistically significant reduction of the number of junctions (Figure 5; Figure S5 C). Most of the observed junctional complexes appeared as disorganized electron-dense areas (data not shown). Noteworthy mainly MOb and MOc morphant ECs are characterized by digitiform extensions at endothelial cell contacts (Figure 5 C′, C″, D; Figure S5 B). These protrusions of the EC membrane, referred to as *filopodia* in literature, are transitional structures that normally characterize AJs before the establishment of the definitive adhesive complex and retract after the maturation of the junction [30]–[32]. Our analysis highlights that controls are totally devoid of these digitiform EC-EC contacts at this developmental stage. Moreover, z*ve-ptp* morphants presented a three-fold decrease in the percentage of EC-EC borders showing well-organized junctional complexes, which is highly significant (p<0.001), with respect to controls (Figure 5 D; Figure S5).

**Figure 6:**
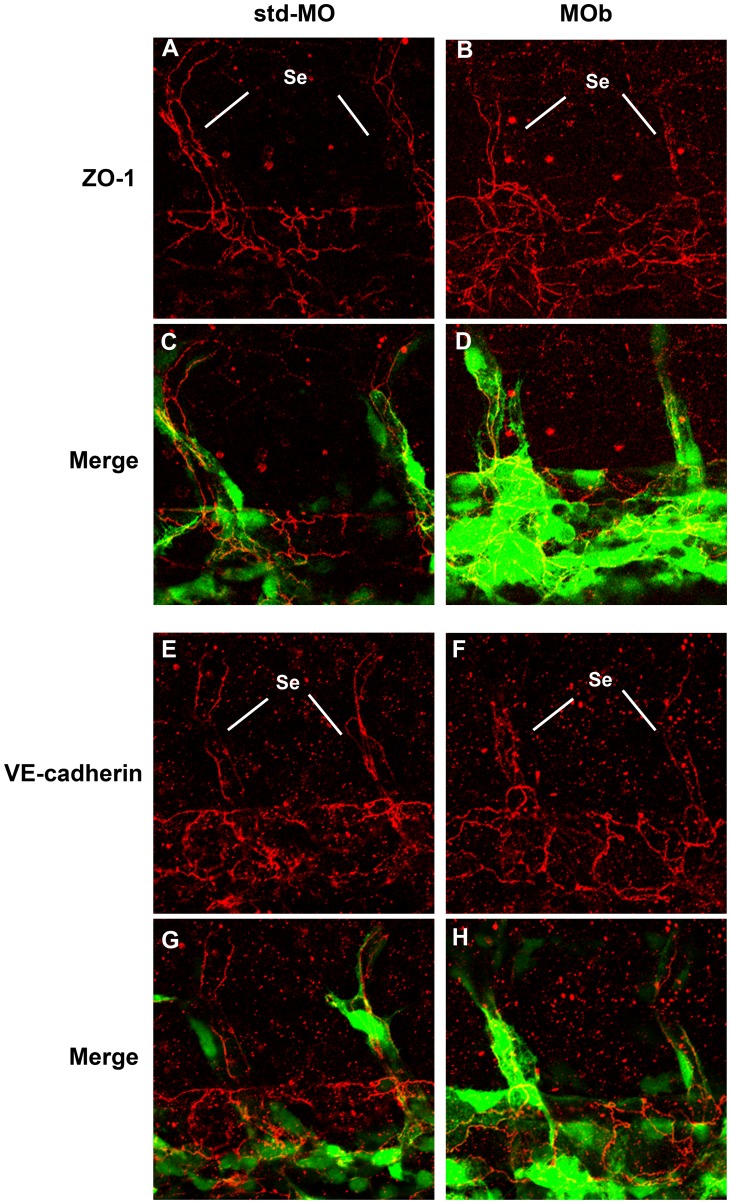
Immunofluorescence analysis by confocal microscopy of endothelial intercellular junctions. Immunofluorescence experiments were performed on tg(*fli1*:EGFP)^y1^ embryos at 2 dpf. Confocal images of tail vessels of std-MO (A, E) and MOb injected embryos (B, F) labeled with an anti-ZO-1 and VE-cadherin antibody (red) and the respective merged images (C, D, G, H). Se: intersegmental vessels.

We also performed immunofluorescence experiments in order to investigate the expression levels and the distribution of VE-cadherin and ZO-1, a tight junction marker. As Blum and colleagues described, these two proteins are localized at EC-EC borders [33]. Whole-mount immunofluorescence experiments showed that the expression of ZO-1 and VE-cadherin in z*ve-ptp* MOb morphants and control embryos at 2 dpf is comparable. This indicates that the expression of two important junctional proteins in adherens and tight junctions is not affected by z*ve-ptp* MOb injection, though their overall distribution along the cell borders appeared different with respect to controls probably due to an altered EC shape (Figure 6). In order to understand whether knockdown of VE-PTP could alter endothelial cell-cell junction organization, we studied human umbilical vein endothelial cells (HUVECs) upon treatment with VE-PTP siRNA. As reported in Figure S6, we found that the distribution of different proteins involved in adherens junction (VE-cadherin, p120) and tight junction organization (ZO-1, claudin 5) was not affected by silencing of VE-PTP. We even detected a slight increase in expression of some of these proteins after VE-PTP knockdown as shown in Western blot assays (Figure S6 B).

VE-cadherin phosphorylation in Y658 was strongly increased in absence of VE-PTP (Figure S6 A). Phosphorylation of this tyrosine has been related to increase in VE-cadherin internalization and turn over.

These data highlight a potential novel role of zVe-ptp in the maturation of EC junctions, corroborating the importance of this protein in junction complex stability and integrity.

## Discussion

Endothelial junction integrity is essential for the maintenance of the physiological role of the endothelium. Vascular permeability is strongly modulated by transmembrane proteins that control interendothelial adhesions. Ve-ptp is the first known tyrosine phosphatase adhesion protein that is only expressed in endothelial cells [18]–[20]. The analysis of mutant and null-mice phenotypes suggested that Ve-ptp is essential for angiogenic processes and vascular system remodeling [18], [19]. Furthermore a recent paper showed in mouse the importance of Ve-ptp/VE-cadherin association in the stabilization of endothelial cell contacts [26], but no thorough investigation has been published so far on the structural role played by this protein in endothelial cell junctions' maturation *in vivo*. We decided to characterize the role of Ve-ptp in the developing vascular system in zebrafish. The structure of the z*ve-ptp* gene product is very similar to mouse Ve-ptp, suggesting that Ve-ptp is a receptor-type protein with a tyrosine phosphatase activity also in zebrafish. z*ve-ptp* showed a selective expression in the endothelium during embryonic development, such as described in mouse. The injection of the first tested morpholino (MOa), which blocks Ve-ptp translation, suggested an evolutionary conserved role of Ve-ptp in angiogenic/remodeling processes.

The injection of two different splice-blocking MOs (MOb and MOc), designed on two different intron/exon junctions, produced altered forms of z*ve-ptp* transcript. We suppose that the obtained gene products lack only part of the extracellular domain, responsible for the interaction with VE-cadherin [21], without putatively altering the catalytic domain. RT-PCR performed to test the efficacy of splice-blocking MOb showed that only a fraction of the z*ve-ptp* mRNA is incorrectly spliced due to the steric action of the splice-blocking MO. We speculate that the minor aberrant transcript might give rise to an aberrant protein that could be incorporated in plasma membrane, although we cannot formally prove it due to a lack of specific antibodies against the zebrafish Ve-ptp protein. To demonstrate the specificity of phenotypes obtained by the MOb injection, we designed a second splice-blocking MO (MOc) targeting the extracellular domain. Phenotypes were qualitatively comparable indicating that failings in junctions' maturation and permeability defects are linked to the impairment of the extracellular domain rather than the total zVe-ptp knockdown.

z*ve-ptp* MOb and MOc morphants displayed a phenotype which is only partially overlapping with the translation-blocking MO (MOa) as shown in Figure S1 and S2. Splice-morphants presented defects related to a loss of endothelium integrity without gross angiogenic defects, suggesting that even a partial modification of z*ve-ptp* gene product may interfere with the maintenance of an intact endothelium. Half of z*ve-ptp* MOb morphants presented blood cell accumulations into the CV plexus that could be due to the extravasation of blood fluid out of the vein. However, only the z*ve-ptp* MOb morphants that are characterized by the most severe phenotype presented blood elements outside of the vein, indicative of a severe impairment of endothelial cell-cell integrity.

The lack of an overt cell integrity phenotype in most z*ve-ptp* splice-morphants prompted us to analyze vascular permeability. The injection into zebrafish blood flow of a fluorescent dye confirmed *in vivo* an increased vascular permeability. By microangiography experiments we detected an increased extravasation of the dye out of the CV plexus and the DLAVs in z*ve-ptp* MOb and MOc morphants with respect to controls (80% vs 38% extravasation in MOb injected embryos and 61% vs 37% in MOc injected embryos, respectively). We performed microangiography experiments only in morphants that showed blood circulation. The vast majority of MOa injected embryos displayed circulatory defects and so they have not been included in this analysis. Moreover z*ve-ptp* MOa morphants *in vivo* did not show phenotypes referable to an altered vascular permeability (such as head haemorrhages and/or vessel blood stases). Consequently, we did not notice a significant percentage of MOa morphants with dye extravasation within the few circulating embryos that could be analysed by microangiography.

Leaks in the vessel wall could be related to a reduction or instability of cell-cell adhesion at the level of the AJs. We suppose that, in our z*ve-ptp* MOb and MOc morphants, if the aberrant zVe-ptp protein lacking part of the extracellular region is incorporated in the plasma membrane, its interaction with VE-cadherin might be disturbed. This hypothesis is supported by the association demonstrated *in vitro*, at the level of the extracellular domains, between Ve-ptp and VE-cadherin, which is accompanied by the enhancement of the adhesive function of the latter, as Nawroth and colleagues showed in transfected cells [21]. These studies have demonstrated that the conformation or the clustering of VE-cadherin, affected by the physical interaction with Ve-ptp, influence its adhesive activity and consequently cell layer permeability [21]. Furthermore, other *in vitro* evidences showed that the adhesive function of VE-cadherin is strongly reduced by the silencing of Ve-ptp expression in ECs [22]. A recent paper showed *in vivo* the effects on vascular integrity and permeability of the zebrafish VE-cadherin knockdown [34]. z*VE-cadherin* morphants presented head haemorrhages and permeability defects similar to the phenotypes we observed in our z*ve-ptp* MOb and MOc morphants.

The ultrastructural analyses revealed a drastic reduction and disorganization of junctions in all z*ve-ptp* morphants ECs (MOa, MOb and MOc injected embryos). The most striking feature of ECs in z*ve-ptp* morphants at 2 dpf, in contrast to controls ECs at the same stage, is the presence of digitiform protrusions at EC-EC contacts. These plasma-membrane extensions are normally transitional structures essential to initiate a cell-cell contact. When cadherins establish mature AJs, the protrusions retract, and the cell-cell contact appears as a closed zipper [30]–[32]. Immunofluorescence experiments showed that VE-cadherin and ZO-1 were present in EC borders of z*ve-ptp* morphants. In order to get some mechanistic insights into the vascular alterations observed in z*ve-ptp* morphants, we switched to an endothelial cell culture system. Immunofluorescence staining of different markers showed that in HUVEC the silencing of VE-PTP expression did not alter adherens or tight junction architecture. However, VE-cadherin phosphorylation, an effect that was linked to dissociation of p120, was significantly increased. Although we could not observe a significant redistribution of p120 from cell-cell junctions, VE-cadherin phosphorylation may be related to the vascular fragility observed *in vivo* in the present paper. Tyr-658 plays a critical role in the regulation of VE-cadherin function, in fact phosphorylation at this site led to the inhibition of cell-cell barrier function [35].

Therefore, we speculate that endothelial AJs in our z*ve-ptp* morphants present an immature structure in which VE-cadherins could be unable to contact each other in order to form the correct and mature adhesion complex. This is an exciting hypothesis that remains to be further investigated. With this aim, since high phosphorylation levels promote the junctional disassembly and the opening of a paracellular pathway [12]–[17], it could be interesting to evaluate the expression level of phospho VE-cadherin also in our z*ve-ptp* morphants. Unfortunately, at present, a specific anti-phospho VE-cadherin antibody that works in zebrafish is not available.

In conclusion, zebrafish Ve-ptp is a specific vascular endothelial PTP. The phenotypes observed in z*ve-ptp* morphants, supported by microangiographies and ultrastructural analysis, suggest a reduction in AJs stability that is reflected in integrity and permeability defects. Our data suggest that Ve-ptp could play a novel critical role in junctional complex maturation, an important event for permeability modulation and for correct vascular network formation.

## Materials and Methods

### Ethics statement

Current Italian national rules: no approval needs to be given for research on zebrafish embryos. Fish were maintained/raised according to EU regulations on laboratory animals (Directive 2010/63/EU).

### Zebrafish lines and maintenance

Zebrafish (*Danio rerio*) embryos obtained from natural spawning were raised and maintained according to established techniques [36]. Embryos were staged according to morphological criteria [37]. Beginning from 24 hpf, embryos were cultured in fish water containing 0.003% PTU (1-phenyl-2-thiourea; SIGMA) to prevent pigmentation and 0.01% methylene blue to prevent fungal growth.

The following lines were used: AB (obtained from the Wilson lab, University College London, London, United Kingdom) and tg(*fli1*:EGFP)^y1^ [29] (from the Lawson lab, University of Massachusetts Medical School, Boston, USA).

### z*ve-ptp* identification

We screened a zebrafish EST (Expressed Sequence Tag) database (http://image.llnl.gov/using) using the mouse *Ve-ptp* nucleotide sequence (gi: 23618913) as a query. We identified an EST (clone IMAGE 4728598) associated with a 3,4 kb cDNA cloned into the pBK-CMV vector, that contains the 3′ UTR and part of the coding sequence, encoding the catalytic domain (PTP domain), the transmembrane region and the last 6 fibronectin type III-like repeats (FN3).

We then used the partial cDNA sequence to screen the zebrafish genomic database (www.ensembl.org) and by several 5′ RACE steps on total RNA extracted from 26 hpf stage embryos, we assembled the entire zebrafish *ve-ptp* (z*ve-ptp*) cDNA sequence (GenBank FJ435363).

5′ RACE was carried out using 5′ RACE System for Rapid Amplification of cDNA Ends (Invitrogen) following manufacturer's instructions.

NCBI (http://www.ncbi.nlm.nih.gov/BLAST/), ClustalW (http://www.ebi.ac.uk/Tools/clustalw/), SMART (http://smart.embl-heidelberg.de/) server's tools and VECTOR NTI version 10.0 (Invitrogen) program were used for basic handling and analyses of nucleotide sequence and protein data. Additional information can be made available on request.

### Expression pattern analysis

RT-PCR (Reverse Transcription-Polymerase Chain Reaction) was performed on total RNAs prepared from zebrafish oocytes and embryos at different developmental stages using the Totally RNA Isolation Kit (Ambion) or the RNAgents Total RNA Isolation System (Promega, Madison, WI, USA), treated with DNase I RNase free (Roche, Basel, Switzerland) to avoid possible contamination from genomic DNA and then reverse transcribed using the ImProm-II Reverse Transcription System (Promega) and Random primers according to manufacturers' instructions.

The cDNAs were then subjected to PCR amplification using GOTaq polymerase (Promega) following the manufacturer's instructions.

The following primers were used for PCR reactions:

*4F*: 5′ GTGGCCCAGACTTTCTGAAC 3′

*5R*: 5′ TGTGCGGCCGTATATAGAGA 3′

Specific *β*-*actin* primers [38] were used to check cDNA quality and possible genomic contamination.

Whole-mount *in situ* hybridization (WISH) was performed substantially as described [39].

For z*ve-ptp* probe preparation, a 1300 bp template (spanning the 7^th^–11^th^ FN3 encoding region) was generated by RT-PCR on total RNA extracted from 26 hpf embryos using the following primers:

*4F*: 5′ GTGGCCCAGACTTTCTGAAC 3′

*6R*: 5′ GTTTGTCCCTCTGCAACGAC 3′

PCR products were cloned into the pCRII-TOPO vector (Invitrogen).

The cDNA-containing plasmid was linearized with *BamHI* enzyme and transcribed with T7 RNA polymerase (Roche) for antisense riboprobe synthesis or linearized with *XhoI* and transcribed with Sp6 RNA polymerase (Roche) to synthesize the sense probe.

Images of stained embryos were taken with a Leica MZFLIII epifluorescence stereomicroscope equipped with a DFC 480 digital camera and IM50 Leica imaging software (Leica, Germany).

For histological sections, stained embryos were re-fixed in 4% PFA, dehydrated, wax embedded, sectioned (8 µm) by a microtome (Leitz 1516) and stained with eosin. Images were taken with an Olympus BH2 microscope (Tokyo, Japan), equipped with a Leica DFC 320 digital camera and the IM50 software (Leica, Germany).

### Morpholino injections and detection of splice variants of z*ve-ptp* transcript by RT-PCR

Three different antisense morpholinos (MOs), MOa (z*ve-ptp* MOa; 5′ TACATTCCGTTGCGTCCACCACCAT 3′), MOb (z*ve-ptp* MOb; 5′ AGGGCTCTGAAACACACACAAACAC 3′) and MOc (z*ve-ptp* MOc; 5′GACCTGGAGATGTGGAAACAAAACA 3′), were synthesized by Gene Tools (Philomath, OR, USA).

MOa was designed on the region surrounding the putative second AUG translation start codon of the transcript lying in a good Kozak consensus context; on the contrary, the first in frame AUG lies in a poor Kozak context, lacking a G at position +4 [40]. MOb and MOc are two splice-blocking MOs designed on the intron 11/exon 12 boundary and intron 12/exon 13 boundary, respectively.

As a control for unspecific effects, each experiment was performed in parallel with a std-MO (standard control oligo) with no target in zebrafish embryos.

MOc was injected in combination with p53 MO (5′ GCGCCATTGCTTTGCAAGAATTG 3′).

All morpholinos were diluted in Danieau solution [41] and injected at 1–2 cells stage. Rhodamine dextran (Molecular Probes) was usually co-injected as a tracer. After injection, embryos were raised in fish water at 28°C and observed up to the stage of interest. For a better observation, the injected embryos were anaesthetized using 0.016% tricaine (Ethyl 3-aminobenzoate methanesulfonate salt, SIGMA) in fish water. Images were acquired and larvae were video-taped by using a Leica MZ FLIII microscope equipped with a Leica DFC 480 digital camera and the IM50 software (Leica, Germany).

At 2 dpf, total RNA was extracted from embryos injected with MOb, MOc and std-MO and from uninjected embryos with RNAgents Total RNA Isolation System (Promega). Reverse transcription was carried out with the ImProm-II Reverse Transcription System (Promega) and random primers according to manufacturers' instructions.

PCR was performed to detect splice variants of z*ve-ptp*.

The following primers were used:

MObFOR2: 5′ TGGTCCTCTGCTGATGGAGA 3′

MObREV2: 5′ CCCCAGGGTCGTGTGATAGT 3′

MOcFORB: 5′ GGCGGATAATGAGCTTAGCA 3′

MOcREVB: 5′ CTCCACGTCTGCACTGTAGC 3′

RT-PCR products were sequenced.

### Microangiography

Microangiography of zebrafish embryos was performed essentially as previously described [42]. tg(*fli1*:EGFP)^y1^ embryos were injected with Dextran-TMR (tetramethylrhodamine; molecular weight 70 kDa, Molecular Probes). Dextran-TMR was dissolved in PBS at 25 mg/ml and microinjected into the sinus venosus of anesthetized zebrafish embryos at 2 dpf.

Specimens were scanned using a Leica TCS NT confocal microscope. For each sample, two images of the fluorescent tracer dye were collected, 10 min (t1) and 15 min (t2) after dye injection. Pictures of the fluorescent vascular tree were also taken. The collected data were processed by ImageJ 1.41o: the pictures obtained at t1 were subtracted to the images obtained at t2, to evaluate changes of fluorescence occurred within 5 min for each injected embryo.

### Histological sections and electron microscopy

2 dpf zebrafish embryos were manually dechorionated and fixed overnight at 4°C with 1.5% glutaraldehyde and 4% paraformaldehyde in 0.1 M sodium cacodylate buffer pH 7.3. They were rinsed in the same buffer and post-fixed for 1 hour in sodium-cacodylate-buffered 1% osmium tetroxide. The samples were then dehydrated in a graded ethanol series, transitioned to propylene oxide and embedded in Epon 812-Araldite. Sections were obtained using a Reichert UltracutE instrument. 0.7–0.8 µm sections were stained with gentian violet and basic fuchsin and photographed with Olympus BH2 microscope (Tokyo, Japan), equipped with a Leica DFC 320 digital camera. Thin sections were cut at 60–70 nm and placed onto copper grids, stained with 2% alcoholic uranyl acetate and aqueous lead citrate and analyzed under a Jeol 100 SX electron microscope.

### Cell culture and Small Interfering RNA (siRNA) experiments

Human umbilical vein endothelial cells (HUVECs) were cultured in MCDB131 medium (Invitrogen) containing 20% FCS, glutamine (2 mM), sodium pyruvate (1 mM), heparin (100 µg/ml, from porcine intestinal mucosa; Sigma), and endothelial cell growth supplement (ECGS) (5 µg/ml, made from calf brain). HUVECs between passage 3 and 5 were used for the experiments. HUVECs were starved in MCDB131 medium (Invitrogen) containing 1% BSA for 3 hrs before proceeding with the experiments. Knockdown of human VE-PTP was established by 48-hrs transfection of cells with the ON-TARGET plus SMART pool siRNAs from Dharmacon and the corresponding non-Targeting siRNAs control. Transfection was performed with LipofectAMINE 2000 (Invitrogen) in accordance with the manufacturer's instructions.

### Immunofluorescence experiments

Embryos at 2 dpf were fixed in 4% PFA 2 hours at room temperature and the immunofluorescence was performed as previously described [33] using mouse anti-human ZO-1, 1∶200 (Zymed) and Alexa fluor 555 goat anti-mouse, 1∶500 (Molecular Probes) and rabbit anti-zebrafish VE-cadherin (CDH-5; from M. Affolter) 1∶500 and Alexa fluor 555 goat anti-rabbit, 1∶500 (Molecular Probes). Images of the tail were taken with a Leica TCS NT confocal microscope equipped with an argon laser.

Cells were fixed and permeabilized with 3% paraformaldehyde, 0.5% Triton X100 (Tx) in PBS for 3 min followed by further 15 min fixation with 3% PFA in PBS (VE-cadherin, PY658 VE-cadherin, p120) or with ice-cold methanol for 5 min (ZO-1 and Claudin5). For immunostaining primary and secondary antibodies were diluted in PBS-5% BSA. Confocal microscopy was performed with a Leica TCS SP2 confocal microscope. For comparison purposes, different sample images of the same antigen were acquired under constant acquisition settings. The following antibodies were used: goat anti-VEC (Santa Cruz), mouse anti-p120 (BD Transduction Laboratories), rabbit anti-ZO-1 (Invitrogen), mouse anti-Claudin5 (Invitrogen), mouse anti-Vinculin (Sigma). Affinity purified rabbit antibody to VE-PTP and to pY658-VE-cadherin were produced and purified by New England Peptide.

### Western blotting

Western blotting has been performed using standard protocols. Protein concentrations were determined using a BCA Protein Assay Kit (Pierce) according to manufacturer's instructions.

### Statistical analysis

Statistical analysis was performed with one-way analysis of variance technique and with Dunnett's post test using GraphPad PRISM version 5.0 (GraphPad, San Diego, California). A p value <0.05 indicates a statistically significant effect.

## Supporting Information

Figure S1 **Vascular and circulatory phenotypes associated to the injection of different amounts of z** ***ve-ptp*** **MOa, z** ***ve-ptp*** **MOb and z** ***ve-ptp*** **MOc.** (A–C) Histograms show the quantification of survival (left panel) and phenotypic classes (right panel) at 2 dpf of tg(*fli1*:EGFP)^y1^ embryos injected with different doses of z*ve-ptp* MOa (A); z*ve-ptp* MOb (B) and z*ve-ptp* MOc (C). Injected embryos were scored for vascular, circulatory and morphological defects at 2 dpf. The dose-response curves of all MOs generate phenotypic classes of increasing severity in a dose dependent manner. “Microhaemorrhages” refers to blood stases in the head region, “Caudal blood stases” refers to blood cell aggregates in the CV plexus, “absent circulation” refers to embryos with no circulating elements and “reduced circulation” refers to a severely reduced blood flow in the trunk/tail region, “Se defects” refers to intersomitic vessels characterized by anomalous branching or truncated, “morphological defects” refers to aberrant morphology of the tail and/or head.
(TIF)

Figure S2 **The z** ***ve-ptp*** **MOa injection caused angiogenic defects at 2 dpf.** (A–D) Confocal images of the tail of tg(*fli1*:EGFP)^y1^ embryos injected with std-MO (A), z*ve-ptp* MOa (B), z*ve-ptp* MOb (C) and z*ve-ptp* MOc (D). Embryos injected with MOa display intersomitic vessels which are either truncated or characterized by anomalous branching (white arrowhead). Se: intersomitic vessels; DLAVs: dorsal longitudinal anastomotic vessels; DA: dorsal aorta; CV: caudal vein. (E–F) The efficiency of splice-blocking is tested by RT-PCR with primers designed on the exons flanking the MO target site. The injection of selected doses of splice-blocking MOs (0.5 pmol/embryo of MOb and co-injection of 0.2 pmol/embryo of MOc with 0.3 pmol/embryo of p53 MO) resulted in the presence of the expected wild-type fragment and the generation of an additional smaller band (red box) corresponding to an aberrant transcript. The sizes of the obtained PCR fragments are indicated. Diagrams in E and F show the position of the *zve-ptp* MOb (MOb; designed on the intron 11/exon 12 boundary), the position of the *zve-ptp* MOc (MOc; designed on the intron 12/exon 13 boundary) and the position of the specific primers (MObFOR2-MObREV2 and MOcFORB-MOcREVB). Boxes represent exons (E11 to E14). The size of each exon is indicated in the respective box.
(TIF)

Figure S3 **z** ***ve-ptp*** **MOc injection gave qualitatively similar results to MOb injection, such as head haemorrhages and blood cell accumulations in the CV at 2 dpf.** Bright-field images of the head and the tail of std-MO (A, B) and z*ve-ptp* MOc injected embryos (C, D). MOc morphants showed small haemorrhages (black arrowhead) in the head (C) and small blood aggregates (red arrows) in CV (D). Anterior to the left. DA: dorsal aorta; CV: caudal vein; black arrowhead: haemorrhage; red arrow: blood aggregates.
(TIF)

Figure S4 **The z** ***ve-ptp*** **MOc injection caused an increase in vascular permeability.** (A–D) Microangiographies were performed on tg(*fli1*:EGFP)^y1^ embryos at 2 dpf by the injection of dextran-TMR (tetramethylrhodamine; molecular weight 70 kDa). All microinjected embryos presented blood circulation. Confocal images of tail vessels of std-MO (A, C) and MOc injected embryos (B, D) at t1 = 10 minutes (A, B) and t2 = 15 minutes (C, D). (E, F) Merge of the images at t2 of embryos injected with std-MO and MOc with the respective images of the tail vessels obtained using tg(*fli1*:EGFP)^y1^ line. Asterisks: dye extravasation. (G) Histogram shows the percentage of embryos injected with MOa, MOb and MOc that showed dye extravasation with respect to std-MO injected embryos.
(TIF)

Figure S5 **Statistical analysis of EC-EC borders in z** ***ve-ptp*** **MOa, MOb and MOc injected embryos.** Quantitative analysis of % EC-EC borders with any type of junctions (A), without junctions but with digitiform EC-EC contacts (B) and without junctions (C) in controls and in z*ve-ptp* MOa, MOb and MOc morphants. The analysis was performed on the TEM acquired images of trunk and tail regions out of three std-MO and five z*ve-ptp* MOa, seven MOb and five MOc independent injected embryos with a total of 112, 221, 150 and 280 EC-EC borders analyzed respectively. *** p<0.001 vs std-MO; ** p<0.01 vs std-MO.
(TIF)

Figure S6 **siRNA knockdown of VE-PTP did not cause alterations of both AJ and TJ architecture.** Immunofluorescence (A) and Western blot (B) analyses of the expression of the major components of both AJs and TJs in HUVEC transfected with non-Targeting or VE-PTP siRNAs. Scale bar: 20 µm.
(TIFF)

Movie S1 **Circulation in a 2 dpf control embryo injected with std-MO.** Only caudal region is shown. Circulation can be seen in the main axial vessels. Anterior to the left.
(AVI)

Movie S2 **Circulation in a 2 dpf morphant injected with z** ***ve-ptp*** **MOb.** Only caudal region is shown. Circulation can be seen in the main axial vessels. Blood cell aggregates can be seen in the caudal vein plexus. Anterior to the left.
(AVI)
