# Supplementary figures and images for: Ve-ptp Modulates Vascular Integrity by Promoting Adherens Junction Maturation
Source: PLoS One. 2012 Dec 14;7(12):e51245. doi: 10.1371/journal.pone.0051245 (PMC3522677; doi:10.1371/journal.pone.0051245)

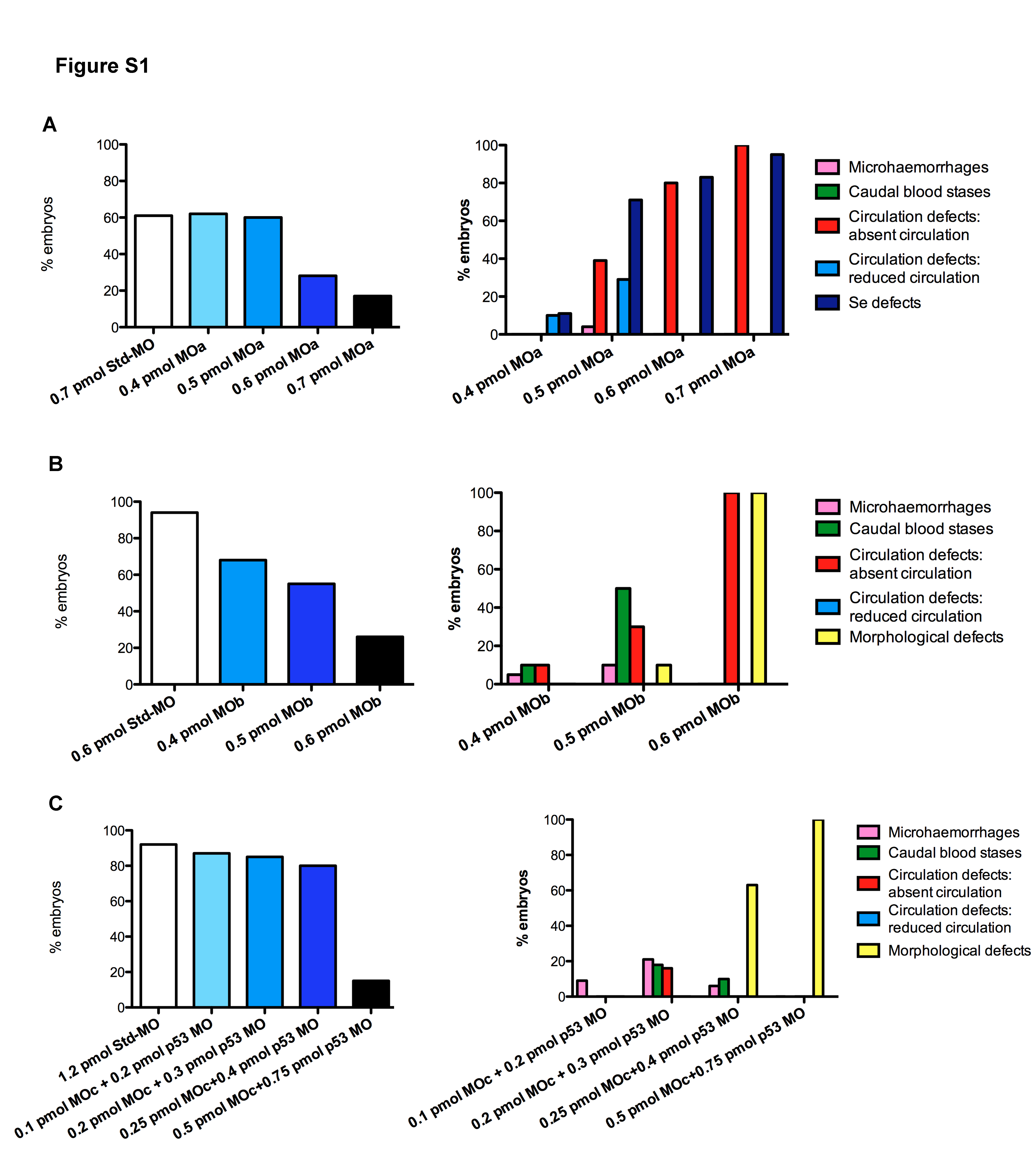

Supplement: Figure S1 — Vascular and circulatory phenotypes associated to the injection of different amounts of z ve-ptp MOa, z ve-ptp MOb and z ve-ptp MOc. (A–C) Histograms show the quantification of survival (left panel) and phenotypic classes (right panel) at 2 dpf of tg(fli1:EGFP)y1 embryos injected with different doses of zve-ptp MOa (A); zve-ptp MOb (B) and zve-ptp MOc (C). Injected embryos were scored for vascular, circulatory and morphological defects at 2 dpf. The dose-response curves of all MOs generate phenotypic classes of increasing severity in a dose dependent manner. “Microhaemorrhages” refers to blood stases in the head region, “Caudal blood stases” refers to blood cell aggregates in the CV plexus, “absent circulation” refers to embryos with no circulating elements and “reduced circulation” refers to a severely reduced blood flow in the trunk/tail region, “Se defects” refers to intersomitic vessels characterized by anomalous branching or truncated, “morphological defects” refers to aberrant morphology of the tail and/or head. (TIF) [file pone.0051245.s001.tif]

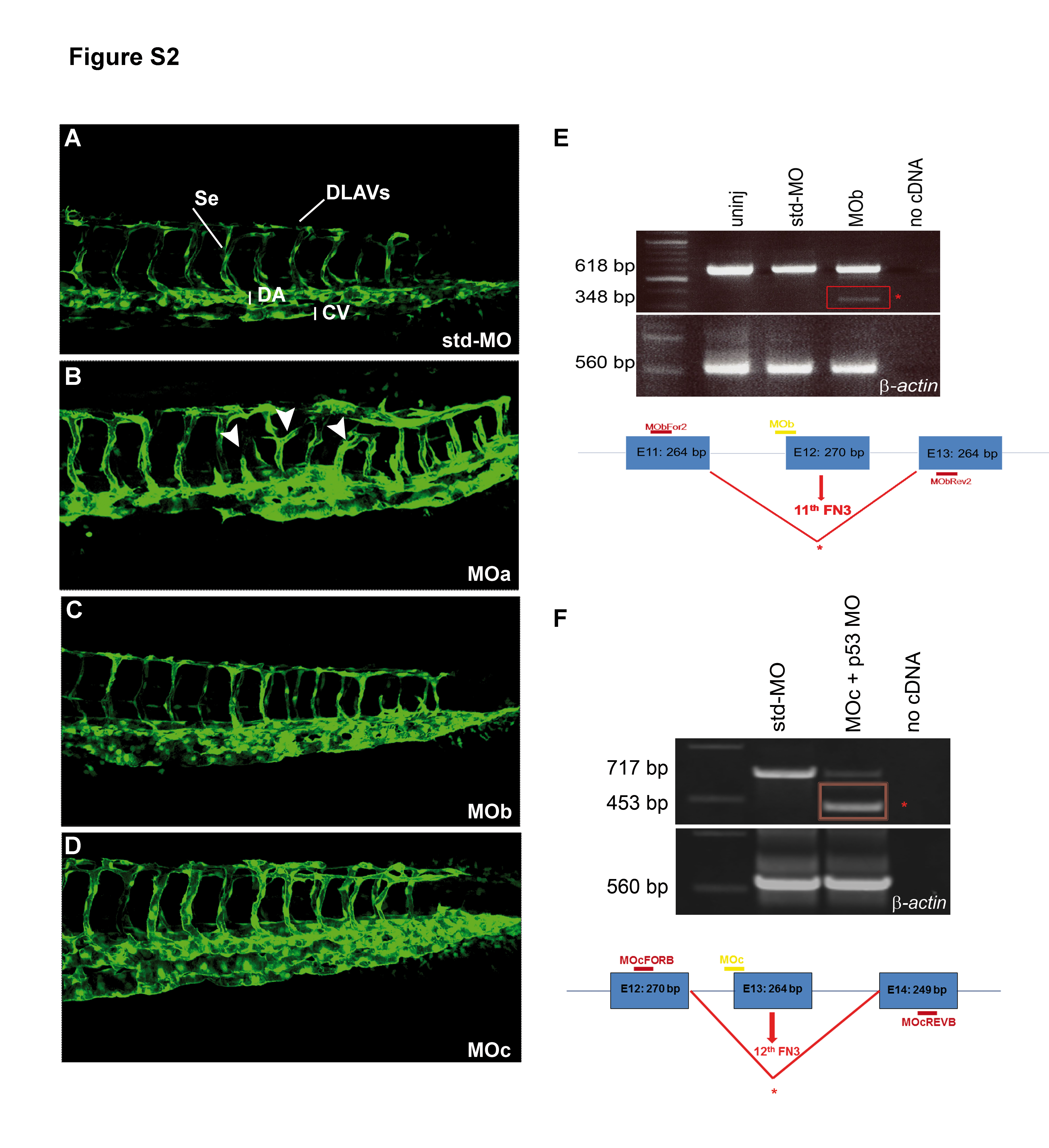

Supplement: Figure S2 — The z ve-ptp MOa injection caused angiogenic defects at 2 dpf. (A–D) Confocal images of the tail of tg(fli1:EGFP)y1 embryos injected with std-MO (A), zve-ptp MOa (B), zve-ptp MOb (C) and zve-ptp MOc (D). Embryos injected with MOa display intersomitic vessels which are either truncated or characterized by anomalous branching (white arrowhead). Se: intersomitic vessels; DLAVs: dorsal longitudinal anastomotic vessels; DA: dorsal aorta; CV: caudal vein. (E–F) The efficiency of splice-blocking is tested by RT-PCR with primers designed on the exons flanking the MO target site. The injection of selected doses of splice-blocking MOs (0.5 pmol/embryo of MOb and co-injection of 0.2 pmol/embryo of MOc with 0.3 pmol/embryo of p53 MO) resulted in the presence of the expected wild-type fragment and the generation of an additional smaller band (red box) corresponding to an aberrant transcript. The sizes of the obtained PCR fragments are indicated. Diagrams in E and F show the position of the zve-ptp MOb (MOb; designed on the intron 11/exon 12 boundary), the position of the zve-ptp MOc (MOc; designed on the intron 12/exon 13 boundary) and the position of the specific primers (MObFOR2-MObREV2 and MOcFORB-MOcREVB). Boxes represent exons (E11 to E14). The size of each exon is indicated in the respective box. (TIF) [file pone.0051245.s002.tif]

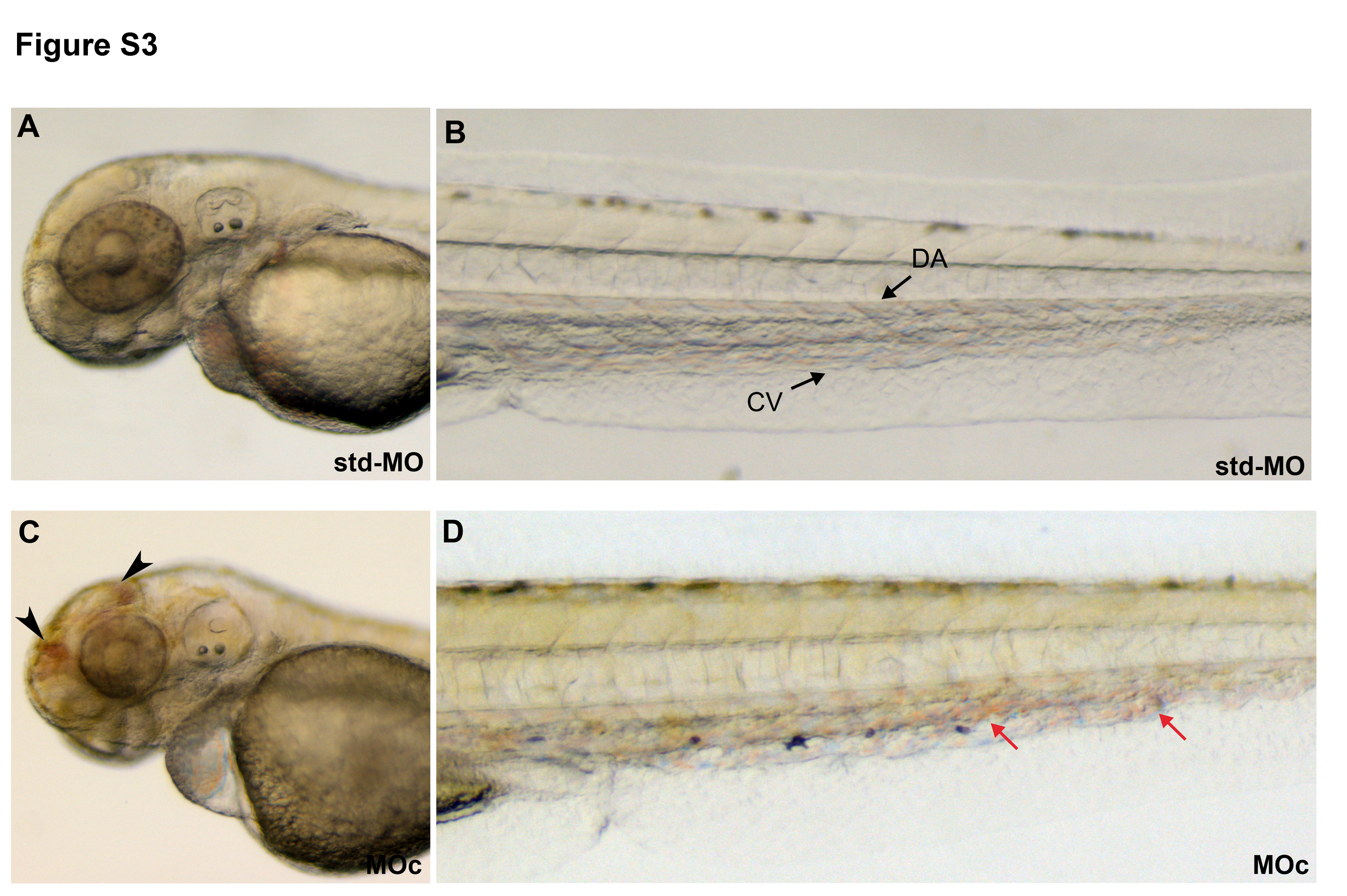

Supplement: Figure S3 — z ve-ptp MOc injection gave qualitatively similar results to MOb injection, such as head haemorrhages and blood cell accumulations in the CV at 2 dpf. Bright-field images of the head and the tail of std-MO (A, B) and zve-ptp MOc injected embryos (C, D). MOc morphants showed small haemorrhages (black arrowhead) in the head (C) and small blood aggregates (red arrows) in CV (D). Anterior to the left. DA: dorsal aorta; CV: caudal vein; black arrowhead: haemorrhage; red arrow: blood aggregates. (TIF) [file pone.0051245.s003.tif]

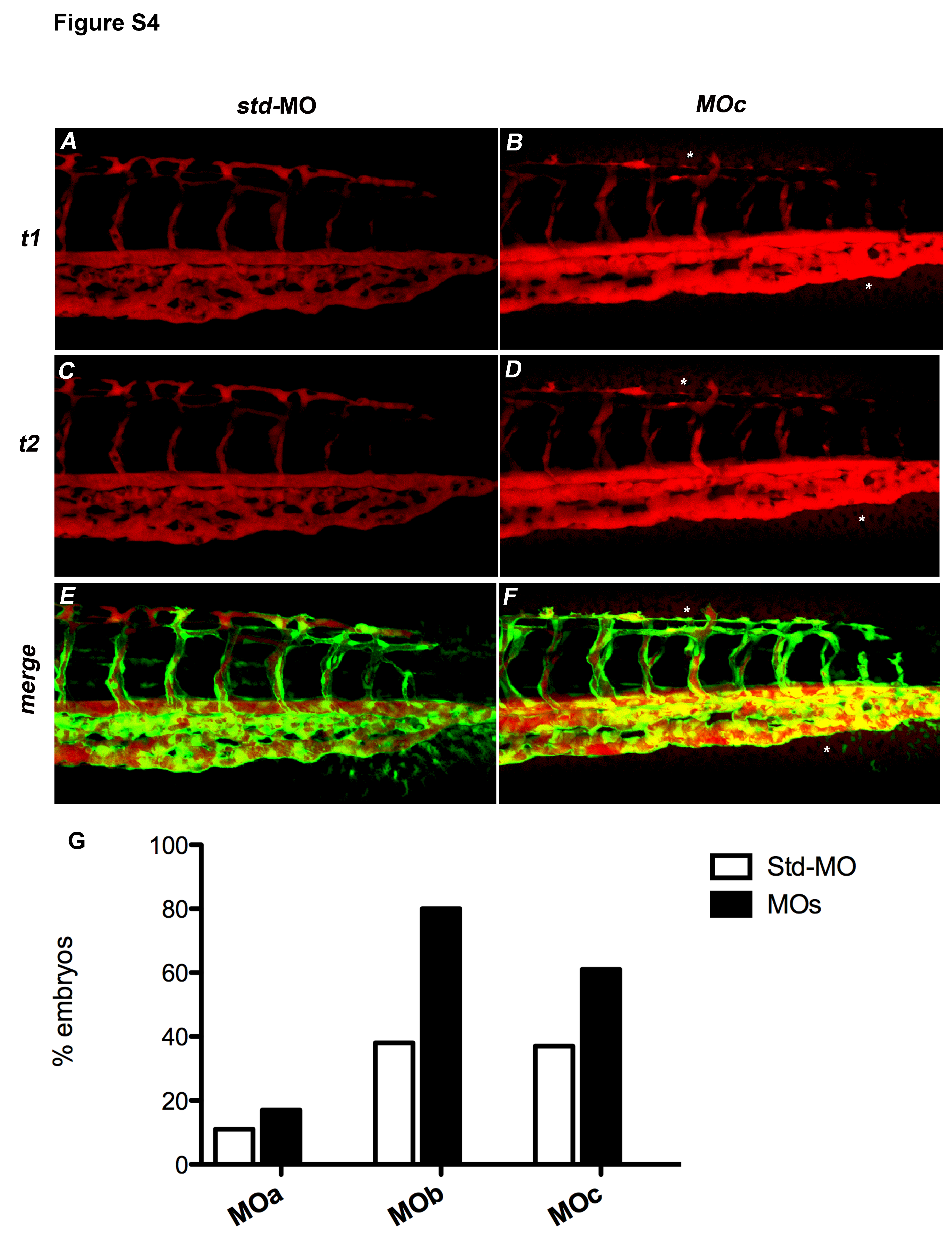

Supplement: Figure S4 — The z ve-ptp MOc injection caused an increase in vascular permeability. (A–D) Microangiographies were performed on tg(fli1:EGFP)y1 embryos at 2 dpf by the injection of dextran-TMR (tetramethylrhodamine; molecular weight 70 kDa). All microinjected embryos presented blood circulation. Confocal images of tail vessels of std-MO (A, C) and MOc injected embryos (B, D) at t1 = 10 minutes (A, B) and t2 = 15 minutes (C, D). (E, F) Merge of the images at t2 of embryos injected with std-MO and MOc with the respective images of the tail vessels obtained using tg(fli1:EGFP)y1 line. Asterisks: dye extravasation. (G) Histogram shows the percentage of embryos injected with MOa, MOb and MOc that showed dye extravasation with respect to std-MO injected embryos. (TIF) [file pone.0051245.s004.tif]

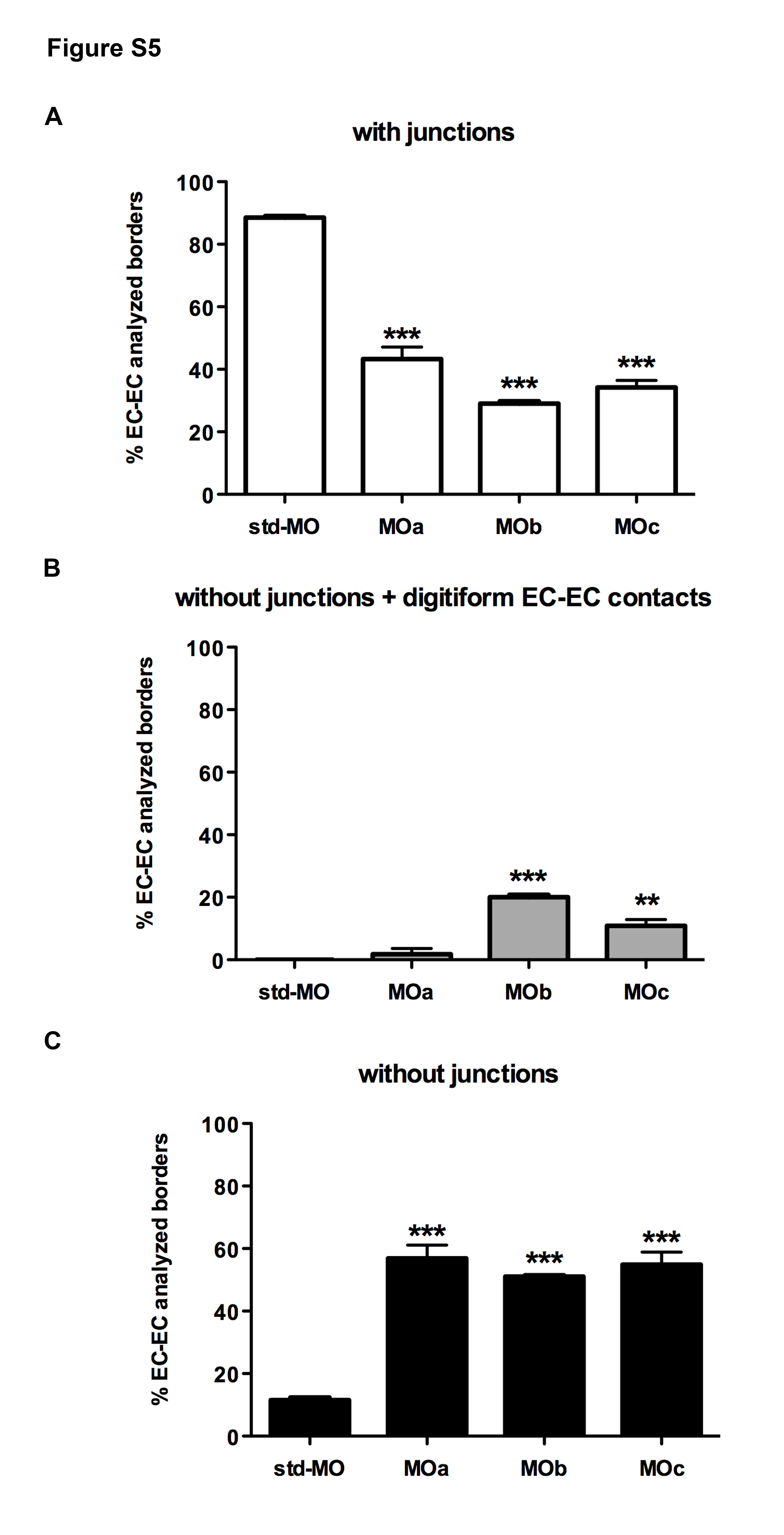

Supplement: Figure S5 — Statistical analysis of EC-EC borders in z ve-ptp MOa, MOb and MOc injected embryos. Quantitative analysis of % EC-EC borders with any type of junctions (A), without junctions but with digitiform EC-EC contacts (B) and without junctions (C) in controls and in zve-ptp MOa, MOb and MOc morphants. The analysis was performed on the TEM acquired images of trunk and tail regions out of three std-MO and five zve-ptp MOa, seven MOb and five MOc independent injected embryos with a total of 112, 221, 150 and 280 EC-EC borders analyzed respectively. *** p<0.001 vs std-MO; ** p<0.01 vs std-MO. (TIF) [file pone.0051245.s005.tif]

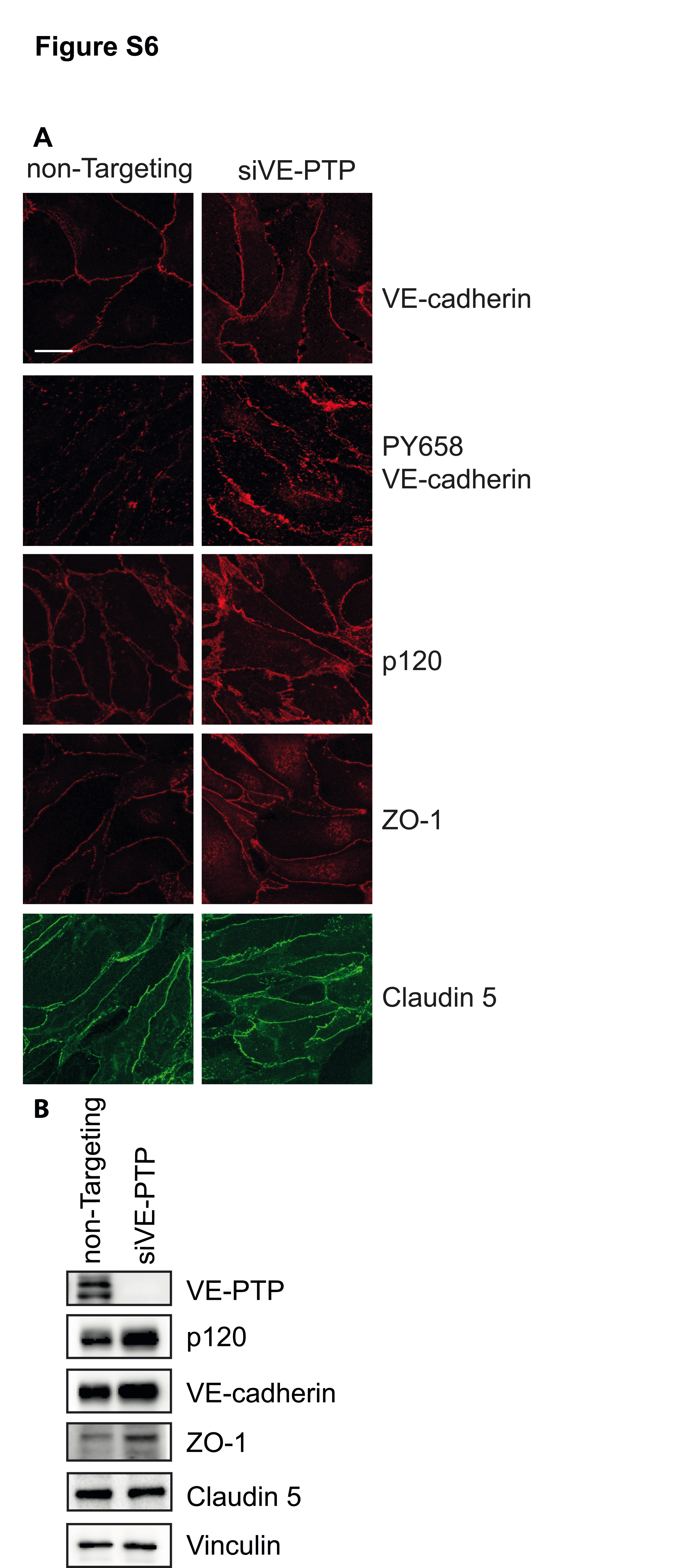

Supplement: Figure S6 — siRNA knockdown of VE-PTP did not cause alterations of both AJ and TJ architecture. Immunofluorescence (A) and Western blot (B) analyses of the expression of the major components of both AJs and TJs in HUVEC transfected with non-Targeting or VE-PTP siRNAs. Scale bar: 20 µm. (TIFF) [file pone.0051245.s006.tiff]
